# Mutational profiling of kinases in glioblastoma

**DOI:** 10.1186/1471-2407-14-718

**Published:** 2014-09-26

**Authors:** Fonnet E Bleeker, Simona Lamba, Carlo Zanon, Remco J Molenaar, Theo JM Hulsebos, Dirk Troost, Angela A van Tilborg, W Peter Vandertop, Sieger Leenstra, Cornelis JF van Noorden, Alberto Bardelli

**Affiliations:** Department of Oncology, University of Torino, SP 142, Km 3.95, Candiolo, Torino, 10060, Italy, Candiolo Cancer Institute – FPO, IRCCS, Candiolo, Torino, Italy; Neurosurgical Center Amsterdam, Location Academic Medical Center, Meibergdreef 9, 1105 AZ Amsterdam, The Netherlands; Department of Clinical Genetics, Academic Medical Center and University of Amsterdam, Meibergdreef 9, 1105 AZ Amsterdam, The Netherlands; Neuroblastoma Laboratory, Pediatric Research Institute, Fondazione Città della Speranza, Corso Stati Uniti 4, 35127 Padua, Italy; Department of Cell Biology and Histology, Academic Medical Center, University of Amsterdam, Meibergdreef 9, 1105 AZ Amsterdam, The Netherlands; Department of Neurogenetics, Academic Medical Center, University of Amsterdam, Meibergdreef 9, 1105 AZ Amsterdam, The Netherlands; Department of Neuropathology, Academic Medical Center, University of Amsterdam, Meibergdreef 9, 1105 AZ Amsterdam, The Netherlands; Department of Pathology, UMC St. Radboud, Geert Grooteplein-Zuid 10, 6525 GA Nijmegen, The Netherlands; Neurosurgical Center Amsterdam, Location Vrije Universiteit Medical Center, De Boelelaan 1117, 1081 HZ Amsterdam, The Netherlands; Department of Neurosurgery, St. Elisabeth Ziekenhuis, Hilvarenbeekse Weg 60, 5022 GC Tilburg, The Netherlands; Department of Neurosurgery, Erasmus Medical Center, ’s-Gravendijkwal 230, 3015 CE Rotterdam, The Netherlands; FIRC Institute of Molecular Oncology, Via Adamello 16, 20139 Milan, Italy

**Keywords:** Glioblastoma, Kinase, Gene, Molecular, Mutation, PI3K-AKT

## Abstract

**Background:**

Glioblastoma is a highly malignant brain tumor for which no cure is available. To identify new therapeutic targets, we performed a mutation analysis of kinase genes in glioblastoma.

**Methods:**

Database mining and a literature search identified 76 kinases that have been found to be mutated at least twice in multiple cancer types before. Among those we selected 34 kinase genes for mutation analysis. We also included *IDH1*, *IDH2*, *PTEN*, *TP53* and *NRAS*, genes that are known to be mutated at considerable frequencies in glioblastoma. In total, 174 exons of 39 genes in 113 glioblastoma samples from 109 patients and 16 high-grade glioma (HGG) cell lines were sequenced.

**Results:**

Our mutation analysis led to the identification of 148 non-synonymous somatic mutations, of which 25 have not been reported before in glioblastoma. Somatic mutations were found in *TP53*, *PTEN*, *IDH1*, *PIK3CA*, *EGFR*, *BRAF*, *EPHA3*, *NRAS*, *TGFBR2*, *FLT3* and *RPS6KC1*. Mapping the mutated genes into known signaling pathways revealed that the large majority of them plays a central role in the PI3K-AKT pathway.

**Conclusions:**

The knowledge that at least 50% of glioblastoma tumors display mutational activation of the PI3K-AKT pathway should offer new opportunities for the rational development of therapeutic approaches for glioblastomas. However, due to the development of resistance mechanisms, kinase inhibition studies targeting the PI3K-AKT pathway for relapsing glioblastoma have mostly failed thus far. Other therapies should be investigated, targeting early events in gliomagenesis that involve both kinases and non-kinases.

**Electronic supplementary material:**

The online version of this article (doi:10.1186/1471-2407-14-718) contains supplementary material, which is available to authorized users.

## Background

Cancer is a multi-step polygenic disease, caused by accumulation of genetic alterations in oncogenes and/or tumor suppressor genes resulting in neoplastic transformation. After the first transforming somatic mutation was found in the *HRAS* gene in human bladder cancer [1], transforming somatic mutations have been identified in numerous genes and in various types of malignant tumors. In the last decade, sequencing of the human genome and development of high-throughput technologies have enabled the systematic analysis of cancer genomes [2–11]. Genes encoding for kinases were found to be overrepresented in the group of cancer genes that have been found to be mutated [12]. Moreover, kinases represent effective therapeutic targets in various types of cancer [13–19]. The description of 518 protein kinases constituting the ‘kinome’ [20] enabled systematic mutation analysis of kinases in colon cancer [2, 6], and other types of cancer [4], including glioblastoma [5, 21].

Glioblastoma is the most common malignant brain tumor and has a poor prognosis. Therapeutic advances have been made in the past decade with the addition of temozolomide chemotherapy to maximal safe tumor resection and radiotherapy. However, median survival is still limited to only 15 months in optimally treated patients [22, 23], and less than a year in the general population [24]. Therefore, novel therapies are urgently needed. For rational drug design, it is essential to unravel the underlying oncogenic mechanisms of glioblastoma. Different genes have been found to be involved in glioblastoma, by changes in expression, methylation, copy number alterations or mutations. A number of kinases has been known to be involved in glioblastoma by various mechanisms. A well-characterized mutation affects the protein kinase *EGFR* and codes for a truncated constitutively activated form which is known as EGFRvIII. In addition, amplification and overexpression of EGFR are important in glioblastoma [25]. *MET* amplification [26], *PIK3CA* mutations and amplification [7, 10, 11, 26], *ERBB2* mutations [10, 11] and amplification of *CDK4* [11] and *CDK6* [10, 11, 26, 27] have been implicated in glioblastoma. Other kinases are found to be overexpressed in glioblastoma [28], including the kinase WEE1 [29]. The question is whether other kinases play a role as well by mutational activation in glioblastoma. We performed a mutation analysis including 34 kinase genes in 113 glioblastoma tumors and 16 high-grade glioma (HGG) cell lines.

## Methods

### Selection of genes

A search strategy was performed by database mining for kinase mutations in cancer in 2006. This search included the OMIM (Online Mendelian Inheritance in Man) of human genes and genetic disorders [30] and COSMIC (Catalogue of Somatic Mutations in Cancer) [31] databases. In addition, a literature search was performed using the key words ‘kinase*’ and ‘mutation*’ in Pubmed. *In silico*, 217 kinases were identified to be mutated in cancer and 76 have been reported to contain non-synonymous somatic mutations in at least two independent tumor samples in the literature. We selected 34 of these 76 kinases for mutation analysis. Reasons for selecting these kinase genes were 1) they are known to be involved in pathways that play a role in the development of glioblastoma, 2) many mutations in these kinase genes have been reported in other cancer types and/or 3) there are small-molecule drugs available for that kinase target (Table 1). In addition, we included *IDH1*, *IDH2*, *NRAS*, *PTEN* and *TP53*, genes known to be (relatively) frequently mutated in glioblastoma [11]. Specifically, we examined 174 exons in which mutations have been previously described for the following genes: *AKT2*, *ATM*, *ATR*, *BRAF*, *BRD2*, *DDR1*, *DYRK2*, *EGFR*, *EPHA3*, *EPHA5*, *EPHA6*, *EPHB2*, *ERBB2*, *ERBB4*, *FGFR1*, *FGFR2*, *FGFR3*, *FGFR4*, *FLT1*, *FLT3*, *FRAP1*, *IDH1*, *IDH2*, *KDR*, *KIT*, *MAP2K4*, *MET*, *NRAS*, *NTRK2*, *NTRK3*, *PAK4*, *PDGFRA*, *PDPK1*, *PIK3CA*, *PTEN*, *RPS6KC1*, *STK11*, *TGFBR2* and *TP53*. In addition, the complete coding sequence of *AKT1* was sequenced in this tumor set, and mutations were not found, as described previously [32]. Furthermore, the molecular and survival analysis of *IDH1* and *IDH2* were published previously [33, 34].

**Table 1:** **An overview of the 152 somatic mutations identified in 113 human glioblastoma samples and 16 high-grade glioma cell lines**

GBM sample #	IDH1	PTEN	TP53	PIK3CA	***EGFR***	Other mutated genes
1 T	R132H		I162F	H1047R† (CH5132799)		
2 T			R248W			
4 T		IVS5-1G > A				
6 T					**IVS21-5C > A**	
8 T		Y155C	E68fs*54			
9 T	R132H		V122fs*25, R280G	**delE110**		
13 T				**F83S**		
14 T			C135Y, C238Y	E545A† (CH5132799)		
16 T*	R132H		I162F	H1047R		
18 T	R132C		Y220C, G245S			
20 T			C275Y			
21 T		T319fs*2	R248Q			
24 T		K13E				
26 T				*M1V*		
27 T				R88Q		
28 T	R132H		R273C			
29 T			*T125R*			
30 T				E545K† (CH5132799)		
34 T	R132H		P64fs*58			
35 T			R213W			
37 T		**R47K**	E180K, Y220C			
38 T	R132H					
43 T						*BRAF*(V600E)† (sorafenib, vemurafenib)
45 T		G132D				
46 T			*S106R*, *D208Y*			
47 T		Y46*	R273C			
49 T			*IVS8 + 1G > T*			
50 T		*L112P*				
51 T				H1047L† (CH5132799)		
53 T		*IVS3 + 1delGT*				*NRAS*(Q61L)† (MEK162)
55 T		R130*	C176*			
56 T		Q149*	*F134L*			
58 T		D24G				
59 T			R273C			
61 T			S260fs*3			
62 T	R132H		E286G, R306*			
64 T			P152S			
65 T	R132H		**N13del**	G118D		
66 T	R132G		R248W			
68 T			R273C			*BRAF*(K601E)† (sorafenib, vemurafenib)
69 T			R175H			
70 T		S305fs*6				
71 T		*K125E*				
73 T	R132H		R273H			
74 T	R132H					
75 T		*Y46H*	P152L			
76 T					*E866D*† (lapatinib, vandetanib, AEE788)	
78 T			H179D			
79 T	R132H					
81 T	R132H		V157F, R282W	C420R		
83 T						*BRAF*(V600E)
84 T	R132L		Y236N			
87 T		*G127R*	R158H			*FLT3*(A627T)† (crenolanib, midostaurin)
88 T		P248fs*5				
89 T		**I253insSTOP**				
92 T					**L210Q** (cetuximab, panitumumab)	
93 T			R282W	H1047Y† (CH5132799)		
96 T	R132H		*K120E*			
97 T			R248W			
98 T		IVS8-1G > A	R213*			
99 T		R242*	Y220C		*P589L*† (cetuximab, panitumumab)	
101 T		IVS3 + 1G > T				
102 T					A597P† (cetuximab, panitumumab)	
104 T		Y336*				
105 T		*IVS4-1G > C*				
106 T*		*IVS4-1G > C*				
107 T		R130*			G598V† (cetuximab, panitumumab)	
108 T*		R130*			G598V	
109 T			R175H			
111 T				*M1V*		
112 T	R132H		R175H			
113 T	R132H		H168R			
114 T		**I253S**				
115 T*	R132H		H168R			
117 T		P96L				***TGFBR2*** **(A204D)** (LY2157299, LY2424087, TR1)
118 T	R132H		M237I			
**Cell lines**						
Gli6		*R130L*	E336*			
SKMG3			R282W			
T98G		L42R	M237I			
U118		IVS8 + 1G > T	R213Q			
U251MG		E242fs*15	R273H			
U373MG		E242fs*15	R273H			
U87		IVS3 + 1G > T				***EPHA3*** **(K500N)** (KB004), ***RPS6KC1*** (Q741*)
SF126		G129R				
SF-763			R158L			
A58		T319fs*2	R248Q			
A60		K13E				
CCF-STTG1		L112R				
D384			A159V			
GAMG			L265P			
Hs683			R248Q			
IGRG-121		Y225*				

37 samples without mutation in sequenced genes are excluded from this table. Mutations depicted in **bold** are, to our knowledge, novel in cancer, mutations in *italics* have been reported in cancer but are novel in glioblastoma.

*indicates recurrent tumor (16 T is recurrent glioblastoma of 1 T, 106 T is recurrent glioblastoma of 105 T, 108 T is recurrent glioblastoma of 107 T, 115 T is recurrent glioblastoma of 2 T). † denotes a (likely) activating mutation. Known kinase inhibitors for that specific target or kinase region are shown between brackets (only shown at first occurence in table).

### Patients, tumor samples and DNA extraction

One hundred and thirteen fresh frozen glioblastoma samples were obtained from 109 patients from the tumor bank maintained by the Departments of Neurosurgery and Neuropathology at the Academic Medical Center (Amsterdam, The Netherlands). All patients were adults except one (age: 15 years). Both primary and secondary glioblastoma were included in this analysis. Research was performed on “waste” material and stored in a coded fashion. Consent for this project was reviewed and waivered by the Medical Ethics Review Committee of the Academic Medical Center and University of Amsterdam (reference number W14_224 # 14.17.0286). Consent for removal of the tissue and its storage in the tumor bank for research purposes was obtained and documented in the patient’s medical chart. Tumor samples were included only if at least 80% of the sample consisted of cancer cells, as verified by H&E staining. For all tumor samples matched germline DNA from blood samples was available. Matches between germline and tumor DNA were verified for all samples by direct sequencing of 26 single nucleotide polymorphisms (SNPs) at 24 loci (data not shown).

In addition, 16 high-grade glioma cell lines were included: the cell lines CCF-STTG1, Hs683, U87MG, U118MG, U251MG, U373MG, T98G (ATCC, Middlesex, United Kingdom), GAMG (Deutsche Sammlung von Mikroorganismen und Zellkulturen, Braunschweig, Germany), SKMG-3 (a gift of Dr C.Y. Thomas, University of Virginia Division of Hematology/Oncology, Charlottesville, VA), D384MG, SF763 (gifts of Dr M.L. Lamfers, Department of Neurosurgery, VU University, Amsterdam, The Netherlands), SF126 (a gift of Dr C. Van Bree, Department of Radiotherapy, Academic Medical Center) and the xenograft cell line IGRG121 (a gift of Dr B. Geoerger, Institut Gustave Roussy, Villejuif, France). A58, A60 and Gli-6 cell lines were derived from our own laboratory [35, 36]. Genomic DNA was isolated as previously described [21].

### PCR and sequencing details

Polymerase chain reaction (PCR) and sequencing primers were designed using Primer 3 and synthesized by InvitrogenTM (Life Technologies, Paisley, UK). PCR primers were designed to amplify the selected 174 exons and the flanking intron sequences, including splicing donor and acceptor regions of the genes (Additional file 1: Table S1). PCR products were approximately 400 bp in length with multiple overlapping amplimers for larger exons. On each sample, 185 PCRs were performed in 384- and 96-well formats in 5 or 10 μl reaction volumes, respectively. PCR conditions have been published previously [21]. Mutation Surveyor (Softgenetics, State College, PA, USA) was used to analyzed the sequencing data. Over 5,000 nucleotide changes were identified during this initial screening. Changes previously described as SNPs were excluded from further analyses. To ensure that the observed mutations were not PCR or sequencing artifacts, amplicons including non-silent mutations were independently re-amplified and re-sequenced in the corresponding tumors. All verified changes were re-sequenced in parallel with the matched normal DNA from blood samples to distinguish between somatic mutations and SNPs not previously described.

In the present study, a total of 23,865 PCR products, covering 9.5 Mb of tumor genomic DNA, were generated and subjected to direct Sanger sequencing. Over 5,000 nucleotide changes were identified during this initial screening. Changes previously described as SNPs, synonymous changes and intronic changes not predicted to affect splicing were excluded from further analyses. To ensure that the remaining mutations were not PCR or sequencing artefacts, amplicons were independently re-amplified and resequenced in the corresponding tumors. All confirmed changes were resequenced in parallel with the matched normal DNA to distinguish between somatic mutations and SNPs not previously described. We used the COSMIC database to investigate whether mutations found were novel in cancer or glioblastoma.

### Cloning

For cloning of the PCR products the pcDNA™3.3-TOPO® TA Cloning Kit (Invitrogen) was used according to the manufacturer’s guidelines. The TOPO ligation reaction (containing 2 μl of fresh PCR product and 1 μl TOPO vector) was performed for 5 min at room temp. Competent E. coli were transformed with the TOPO cloning reaction and spread on a pre-warmed selective plate (ampicillin). Plates were incubated at 37°C overnight. White colonies were picked for PCR analysis and sequencing, using the protocol described above.

## Results and discussion

Clinical and histological characteristics of 109 glioblastoma patients from which 113 tumor samples were extracted are shown in Table 2. An overview of the 148 somatic mutations that we identified in these 113 human glioblastoma samples and 16 high-grade glioma cell lines is shown in Table 1. Somatic mutations were found in *TP53* (61 mutations), *PTEN* (39), *IDH1* (20), *PIK3CA* (13), *EGFR* (7), *BRAF* (3), *EPHA3* (1), *NRAS* (1), *TGFRB2* (1), *FLT3* (1) and *RPS6KC1* (1). To our knowledge twenty-five of these have not been described before in glioblastoma and are highlighted in Table 1.

**Table 2:** **Baseline characteristics of 113 glioblastoma patients. Data are mean (range), number (%) or median (95% CI)**

Characteristic	Specification	Outcome
Age	Mean (range), in years	54 (15–81)
Irradiation dosage	Mean (range), Gy	39 (0–88)
KPS	Mean (range), in points	76 (50–90)
Gender	Male	61 (56%)
	Female	48 (44%)
Surgical procedure	Gross total removal	62 (57%)
	Biopsy or irradical resection	57 (43%)
Tumor occurrence	Primary glioblastoma	94 (86%)
	Secondary glioblastoma	15 (14%)
	Recurrent tumor	8 (7%)
Overall survival*	Median (95% CI), in days	252 (206–318)
Progression free survival*	Median (95% CI), in days	131 (105–157)

Data are mean (range), number (%) or median (95% CI) *Survival data was available for 98 glioblastoma patients.

*Abbreviations*: Gy, Gray; KPS, Karnofsky Performance Status.

### Overall

The observed mutation rate of all non-synonymous somatic mutations (13.2 mutations/Mb) was higher than the expected ‘passenger’ mutation rate (*P* < 1×10^−15^, binomial distribution) [37], indicating that most of these mutations probably represent ‘driver’ mutations. In the sequenced genes, 76 out of 113 (67%) glioblastoma tumors displayed at least one somatic mutation; no mutation was identified in 37 glioblastoma samples. In all cell lines at least one mutation in *TP53* or *PTEN* was found. The maximum number of mutations in a single sample observed was three, occurring in both tumor and cell line samples. Only non-silent mutations were further investigated to determine whether they were somatic or not. Differences in non-silent mutation rate between untreated samples and recurrent samples treated prior with temozolomide chemotherapy were not found. Therefore, it is impossible to conclude whether samples derived from patients that had been pretreated with temozolomide (n = 8) developed a hypermutator phenotype, as was described for other glioblastoma samples after temozolomide treatment [5]. Remarkably, no additional mutations were observed in the four recurrent tumors compared to their primary glioblastomas, which were both included in the mutation analysis.

**Figure 1:**
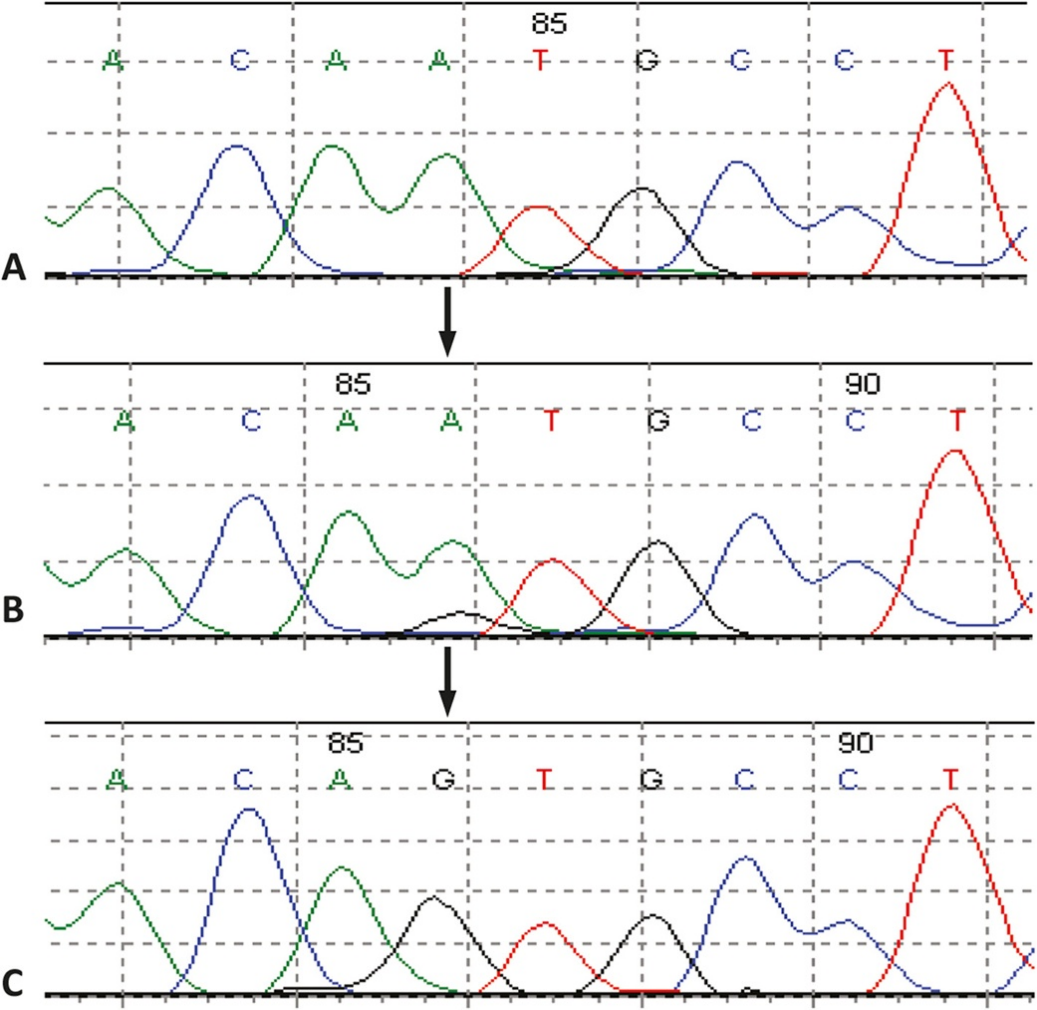
**Somatic mutation confirmed by cloning. A**, chromatogram of matched normal blood sample; **B**, chromatogram of tumor sample; **C**, chromatogram of cloned PCR product. Arrows indicate the location of missense somatic mutations. Numbers above the sequences are part of the software output. *PIK3CA*, c.158A>G, p.M2V.Mutation prevalence of genes.

Some of the mutations were probably present in a small fraction of cancer cells [38, 39]. Cloning of the PCR product helped to confirm the mutation in all tested samples. An example is shown in Figure 1. For some amplicons, the PCR reaction failed twice, as occurred for example for exon 4 of *PTEN* in the SKMG-3 cell line. This cell line is known for a deletion containing exon 4 [40]. Hence, in this case, the incapacity of amplification is probably caused by the deletion.

### Mutation prevalences of genes

For *PIK3CA* and *PTEN* the mutation frequencies are not different from previous reports [41–43]. The mutation frequencies of *TP53* (46%) and *IDH1* (17%) are higher than previously reported in glioblastoma samples [10, 41, 42, 44]. Fourteen % of the samples were from secondary glioblastoma, which is also higher than in the aforementioned studies. Since *TP53* and *IDH1* mutations occur mostly in secondary glioblastoma, the relatively high number of secondary glioblastoma can explain the relatively high number of *TP53* and *IDH1* mutations. Regarding *TP53*, we identified seven samples with two mutations in *TP53*. When corrected for mutated single samples, the mutation percentage is 39%, still slightly higher than reported. Mutation details for *IDH1* have been published separately [34, 45]. In *EGFR*, the mutation frequency is lower than reported previously, due to the fact that we sequenced only exons belonging to the kinase domain, whereas Lee *et al*. found mutations predominantly in the extracellular domain [37, 46]. No *AKT1* mutations were found, as described previously [32].

A new mutation hotspot, providing a novel therapeutic target in a significant percentage of glioblastoma patients, was not identified in the sequenced kinase genes. This may be due to the limited number of kinases which was sequenced in this project. However, other genome-wide glioblastoma sequencing projects have not resulted in the discovery of novel mutation hotspots in kinases either [10, 11, 47]. This supports the theory that every cancer type may have its own mutated cancer candidate genes, and only a few of these genes are shared by different cancer types [48]. Furthermore, the mutations themselves, rather than the genes, may be cancer-specific [9, 10, 44, 45]. Therefore, we cannot exclude that other exons of the genes may exhibit a more frequently mutated genotype. Notably, glioblastomas exhibit a different mutation profile for some genes as compared to other tumor types. For example, most *EGFR* and *ERBB2* mutations in lung cancer are found in the kinase domain [49, 50], and that is why we included those regions in our study. However, recent studies show that these genes are predominantly mutated in the extracellular domain in glioblastoma [10, 46]. Some of the novel mutations that we have found affect kinases, for example *EPHA3*, recently demonstrated as a functional targetable receptor in glioblastoma [51]. These are clearly amenable to pharmacologic intervention and represent potential novel therapeutic targets for glioblastoma.

### Cell lines

Neither *IDH1* nor *PIK3CA* mutations were found in any of the cell lines examined. Compared to the mutation frequency (17% and 11%) that we found in 109 glioblastoma samples, the lack of *IDH1* and *PIK3CA* mutations in our panel of 16 HGG cell lines is remarkable. However, currently available glioblastoma cells lines do not have endogenous *IDH1*/*2* mutations. Thus far, three anaplastic glioma cell lines have been reported to have *IDH1*/*2* mutations [52–55]. However, the fact that there were no mutations in the 16 established cell lines is not surprising, because most lines are derived from glioblastomas and most of these were probably primary glioblastoma, in which *IDH1*/*2* mutations are rare [6, 44, 45]. On the other hand, glioblastoma cell lines with *PIK3CA* mutations have been described [43].

Two cell lines generated from glioblastoma samples included in our mutational screen were also subjected to the mutation analysis we performed. Of note, both one *TP53* (R248Q) and two *PTEN* mutations (T319fs*2 and K13E) in the cell lines were found in homozygosity, whereas the same mutations in the corresponding original tumor were heterozygous. We included tumor samples only if at least 80% of the sample consisted of cancer cells, as verified by H&E staining. Therefore, we considered the chance of contamination by normal brain tissue to be small. As established cell lines derived from glioblastoma resemble the original tumors in patients poorly when compared at the level of DNA alterations [35, 56], we argue that one allele of the gene may have been lost during the establishment of the cell lines or during cell culture afterward.

One of the changes that was identified in *EPHA3* (K500N), was previously reported by us [57], to occur in the cell line U87MG, for which no matched normal tissue is available. Therefore, the somatic status of this mutation could not be ascertained. As the U87MG cell line is widely used in basic glioblastoma research, our results suggest that U87MG may not be a viable model for all research proposes due to the *EPHA3* mutation.

### PIK3CA, PTEN in the PI3K-AKT pathway

Somatic mutations in *PIK3CA* have been found in various tumor types, affecting particularly exons 9 and 20 and to a lesser extent exon 1. In our glioblastoma samples, twelve mutations were found in PIK3CA, five were located in exon 1, two in exon 9 and three in exon 20. One of the five mutations in exon 1 has not been reported before in cancer.

*PIK3CA* and *PTEN* mutations were found mutually exclusive in our glioblastoma samples, as was previously observed in glioblastoma [58, 59], and other tumor lineages [60, 61]. This suggests that the mutations exert overlapping cellular functions. Indeed, both the lipid kinase PI3K and the phosphatase PTEN act as central regulators of the PI3K-AKT pathway by controlling the cellular levels of phosphatidylinositol-3-phosphate. Activating mutations in the *PIK3CA* oncogene result in increased PI3K catalytic activity and constitutive downstream signaling. In contrast, the tumor suppressor protein PTEN counteracts the effect of PI3K and acts as a negative regulator of PI3K signaling [62]. Consequently, inactivating mutations in *PTEN* also result in constitutive downstream signalling of the PI3K-AKT pathway.

In our limited analysis, we found most mutations in genes to belong to the PI3K-AKT pathway; mutational activation of this pathway was observed in at least 50% of glioblastomas, similar to findings in other studies [48, 63]. Whole-genome sequencing efforts also studied non-kinase genes in this pathway (*NF1*) and thus revealed an even higher percentage (~90%) [11]. This indicates that the PI3K-AKT pathway represents an interesting therapeutic target for glioblastomas. However, the results of most clinical trials with (kinase) inhibitors interfering in this pathway have been disappointing thus far [25, 64–66].

As many glioblastoma have an activating *EGFR* mutation [10, 11, 46], the first clinical studies with EGFR inhibitors had high expectations [25]. However, the response to EGFR inhibitors was found to be limited to only 15-20% of glioblastoma patients with activating *EGFR* mutations [42, 67–69]. The partial response is likely caused by other molecular events downstream of EGFR, leading to simultaneous activation of downstream effectors. For example, the oncogenic PI3K-AKT signaling pathway is activated in 15% of glioblastoma via activating mutations in the *PIK3CA* oncogene [11] and in 36% of glioblastoma via mutationally or transcriptionally inactivated *PTEN* [11]. As a result, the limited response of therapeutic EGFR inhibition was thought to be neutralized by loss of *PTEN*. This explains the correlation observed between the response to EGFR inhibitors and the co-expression of EGFRvIII and PTEN proteins [37, 42, 70] or phosphorylated AKT [71]. PTEN-deficient glioblastoma patients were expected to respond to a cocktail of drugs consisting of an EGFR inhibitor and rapamycin [70], but the results were not impressive either [72]. Rational drug design and rationally designed clinical trials to test these drugs are needed, because an almost infinite number of compounds is currently available, and these can be tested in limitless numbers of combinations. With genomics approaches, discoveries of common features of different types of tumors may lead to new therapeutic targets and drugs for other tumor types as well [28, 73, 74].

These findings indicate that single-agent kinase inhibition therapy is not sufficient to target the PI3K-AKT pathway successfully. Similar negative findings have been reported for single drug trials that target the ERK pathway in colon carcinoma [75], where mechanistic studies have shown that concomitant inhibition of other pathways, (i.e. PI3K-AKT) is more effective in these patients [76]. Analogous to such investigations, additional research efforts, such as ours, should pursue the discovery of other targetable molecular alterations in glioblastoma, in order to facilitate the development of multidrug trials that are less likely to fail due to resistance mechanisms. Other kinases were found to be important in glioblastoma as well and may provide therapeutic options. Inhibition of the kinase WEE1 has shown to sensitize glioblastoma to ionizing radiation *in vivo* [29, 77]. Other single-agent kinase therapies targeting PDGFRA, MET and FGFR2/3 should be studied as well [47]. However, the question has been raised whether rational single-agent kinase inhibition treatment will suffice in the treatment of glioblastoma. Multiple pathways are altered in glioblastoma [10, 11, 28] by (epi)genetic [10, 11], transcriptional [78–80] and metabolic mechanisms [10]. An important hallmark of glioblastoma is intratumoral heterogeneity [38]. Thousands of clonal mutations have been identified in glioblastomas, but, only some are common [38], showing that the cancer phenotype iscomplex. Each tumor, and also each glioblastoma, evolves as a result of stochastic and environmental processes in different mutations [39]. As tumor cells contain thousands of mutations, both ‘driver’ and ‘passenger’, that affect many pathways [81], it may be impossible to target these adequately [39]. Notably, the ‘passenger’ mutations, most of the alterations, may not provide growth advantage per se, but could cause resistance to therapy in a subset of cells, which can dominate the tumor next. We, and others [39], are convinced that the focus should be on targeting early common alterations in glioblastoma. For example, inaugural *IDH1* mutations [28], causing metabolic alterations, may be an interesting therapeutic target [52, 82]. As only a subset of glioblastoma has *IDH1* mutations [45], for *IDH1* wild-type tumors other, perhaps metabolic [83, 84], therapies should be investigated.

## Conclusion

In conclusion, molecular profiling of tumor genomes has provided a comprehensive list of cancer genes and of the signaling pathways they control. These efforts have, amongst others, led to the discovery that glioblastomas harbor thousands of mutations whereas only some common driver genes are involved. Extensive whole-genome sequencing of glioblastoma has been performed in recent years [11, 47], but it has been calculated that the discovery of molecular alterations in GBM is nowhere near saturation as of yet [85]. Whereas the present study did not reveal novel mutational hotspots in kinases in glioblastoma, we did observe a strong clustering of mutations in genes belonging to the PI3K-AKT pathway. This pathway is more frequently activated by genomic aberrations than any other signaling pathway in many tumor types. However, due to the development of resistance mechanisms, kinase inhibition studies targeting the PI3K-AKT pathway for relapsing glioblastoma have mostly failed thus far. Other therapies should be investigated on targeting both kinases and non-kinases that are involved in early events in gliomagenesis.

## Electronic supplementary material

Additional file 1: Table S1: Thirty-nine genes selected for mutation analysis and primer details to sequence the indicated 174 exons of the selected genes. Primer sequences are in 5’ to 3’ direction. (DOC 316 KB)
