# Supplementary material for: Mutational profiling of kinases in glioblastoma
Source: BMC Cancer. 2014 Sep 26;14:718. doi: 10.1186/1471-2407-14-718 (PMC4192443; doi:10.1186/1471-2407-14-718)
Supplement: Supplementary file 1 — Additional file 1: Table S1: Thirty-nine genes selected for mutation analysis and primer details to sequence the indicated 174 exons of the selected genes. Primer sequences are in 5’ to 3’ direction. (DOC 316 KB) [file 12885_2014_4907_MOESM1_ESM.doc]

# Additional file 1: Table S1

| **Gene** | **Exon** | **Forward** | **Reverse** | **Sequencing primer** |
| --- | --- | --- | --- | --- |
| ***AKT2*** | **10** | GAGGGTGAGGCTGTGTGTG | GAGGATGGAGGAGAAATGAGG | GGAGGGTTGATGTCCAGGAG |
| ***AKT2*** | **11** | GTGTGAGGCCAAGGGTAGG | TGTGGGGACGACACACTG | GTGTGTTTCCAGCCCAGATG |
| ***ATM*** | **11** | GGACACCAGGTCTTATTCCTTC | ATAAAGCCATCTGGCATCAA | CAAGAATCTTCCCAAATGTAATCA |
| ***ATM*** | **17** | ACAGATGTGAGCCACTGTGC | GAGGCCTCTTATACTGCCAAA | GCCCAGCCTGATTAGGTAAA |
| ***ATM*** | **21** | GCAAGGTGAGTATGTTGGCA | GATCAGCCTACGGGAAAAGA | GGCATATTCCACATAATGACAAA |
| ***ATM*** | **27** | CCTTGTTTGGCTGATTTTCATAC | AGACATTGAAGGTGTCAACCAA | AGTCTACAGGTTGGCTGCATAGA |
| ***ATM*** | **34** | ACGCCTGGCTAATTTTGTATTTT | TGTGTGAAGTATCATTCTCCATGA | CACTCGGCCTTAAGGTTAATTCT |
| ***ATM*** | **35** | TGAATGACTAGTGAAAGTCCTTTGA | CAATTTAACAGTCATGACCCACA | TTTTTCAGTGGAGGTTAACATTCA |
| ***ATM*** | **38** | ATTCCTTCAGAACCAATTTTGTG | ACACTTTGCAACACCTTCACCTA | AGCATAGTGGGAGACAGACACAT |
| ***ATM*** | **42** | TTCCATGTTTTCAGGATCTTCTC | GGAGTATCCCTGAATGTTTAGCC | GGCTGTGTAAATATCCACCAACA |
| ***ATM*** | **47** | CCCTGACAAGTAGTTAAGTCCTCAAT | GACCACATGATGGACTGATAGAA | GTCCTCAATGAATGGTAGTTGCT |
| ***ATM*** | **49** | TGGACAAGTTTGCAATAGTTCAT | CCGACCTTTAGAGCTCAAA | GACCAAGTCACTCTTTCTATGCAA |
| ***ATM*** | **50** | TTTGTCCTTTGATGCTTAGGAAG | TTATGTGTAGAGCACTGGACCAA | TTTCCCTGGGATAAAAACCC |
| ***ATM*** | **52** | CCTCTGCCTTTTTCTCACACAT | AGCCTTGAACCGATTTTAGATG | ACGCTCTACCCACTGCAGTATC |
| ***ATM*** | **54** | CTCAATCAGAGCCTGAACCAC | GAGGTGTAGAACAGAAGGCAATG | GGCCAGTGGTATCTGCTGAC |
| ***ATM*** | **58** | TTCCCTGTCCAGACTGTTAGC | TGATTTAATAATGAAGATGGGTTGG | TTCATCTTTATTGCCCCTATATCTG |
| ***ATR*** | **25** | CCCCTAAGAAGGATACTGGAAAA | TGTGTGCTAGGCATTCAGATAGA | GATTGCAGTAAAGGCAAGGC |
| ***ATR*** | **35** | GTGGCTACAGAGAGCCAACTG | CATGTGCTTTGCCATATAGACTT | TGAATGAATGAGATGAAGCAATG |
| ***ATR*** | **40** | CACAGACTGCTGAACCTTTGTAA | AAACGTTACGGTGAATGTGAATC | TTGCCATCAGTACAAATGAGTTTAG |
| ***ATR*** | **45** | GTAAAAGCAAAGGCAGAGCTACA | CAAACATATGTAGGGGCCAATAA | GCTACATGGCTCCTTCATGTTAC |
| ***BRAF*** | **11** | TTAAGGGGATCTCTTCCTGTATC | CAAAATAGTTTATTGATGCGAACAG | TGTATCCCTCTCAGGCATAAGG |
| ***BRAF*** | **15** | AGCAGGTTATATAGGCTAAATAGAAC | TGATTTTTGTGAATACTGGGAAC | TCTTACCTAAACTCTTCATAATGCTTG |
| ***BRD2*** | **3** | GCACCTGGATTCATCAGACTATT | AGCATCTACACTAGGCAGACCAC | ATTCTTGCTCATCCCACACC |
| ***BRD2*** | **11** | ACAGTGGGAACATACTGGAAGAG | TAAGGTTCCCAAGTTCCCTTCT | CAAACAGACCCCACCATCTT |
| ***DDR1*** | **13** | GAGCTTCACTTTCTCTGCCTGTA | AAGAGAATGGGAAAGGGATACAA | GCCTGTAAGATGGTGCTGATAGT |
| ***DDR1*** | **19** | ACCAGAGCATGGAGAGGAAAG | ACAGGTACACCTGCATTGTGG | CAAGGGAGAGGAGTTGGAAA |
| ***DYRK2*** | **3.1** | GGAACCCTGAATATGTACAGCAA | GCTTCTTAGCATTTAGACCCAAG | ATCACATGTTTTGTGGCCTTTAC |
| ***DYRK2*** | **3.2** | CCCCATGACACCTGAACAAG | TCATGCTCAGCAGCTCAAAC | ACTCACAGCCTTCGAACACC |
| ***DYRK2*** | **3.3** | TCCTCAAGGTCATTGGGAAG | ACGTGTAGACACGCTGATGC | GGCCTACGATCACAAAGTCC |
| ***DYRK2*** | **3.4** | AGAATTTCACCTTCCGCAAC | GCATGCCCAACAGTTCAATC | TTGAGCTGCTGAGCATGAAC |
| ***DYRK2*** | **3.5** | TTAAAGCAGCAGGGTAGAAGC | GGATCATCACACCCCTTCAG | GTTACGAGCATCAGCGTGTC |
| ***DYRK2*** | **3.6** | TTTGTGAGCTCCAAGGGTTATC | GTAAGGCTCAGCAATGTCGTATC | GTTACTGCACTGTCACGACTCTC |
| ***EGFR*** | **15** | AACAGTGACCATATCAAGCAGGT | CTCAAATACAAACCTCGGCAAT | GGTGCAATCACAGAATAACTGGT |
| ***EGFR*** | **18** | CAGAGCCTGTGTTTCTACCAACT | GGAAATATACAGCTTGCAAGGAC | AAGCTCTGTAGAGAAGGCGTACA |
| ***EGFR*** | **19** | GGTAACATCCACCCAGATCACT | CTGTCTCTAAGGGGAGGGAGTTA | CCACTAGAGCTAGAAAGGGAAAGA |
| ***EGFR*** | **20** | GTAAACGTCCCTGTGCTAGGTC | CATGGCAAACTCTTGCTATCC | CATTCATGCGTCTTCACCTG |
| ***EGFR*** | **21** | GAGGAACTGGATGGAGAAAAGTT | TCAGGAAAATGCTGGCTGAC | CCTTTCCATTCTTTGGATCAGTAG |
| ***EGFR*** | **22** | AGACTGAAATCCCCTGTTGC | TTCCTCTGCCGTGTTTTCTC | ATCTGGCTCGTCTGTGTGTG |
| ***EGFR*** | **26** | CCTTCACAATATACCCTCCATGA | CCATGTAAAATAGAGCCATAGTGGA | ATTCAGGAAAAGTGGATGAGATG |
| ***EPHA3*** | **6** | GGGGCCAGATTAATATTCAAGAG | GAAACAGCTTCTTGCCTACACAG | GATAGACTCCATTTGCATGTTCC |
| ***EPHA3*** | **7** | GCCTAAGTTGCGTTCAGAGAATA | TCAGACATCTTTTGCAAGATCC | AGAATCAGGTTTGAGTGGTTCAC |
| ***EPHA3*** | **13** | TTCAGCCTTGTATCCATTTGC | CGTTTTGGGTCATGATTTCTACT | CATATCTTTGTCTTGAGTGCTTAGG |
| ***EPHA3*** | **14** | GCTACTTCTGCTTGGTATTGAAA | ATGTTCCACTTCCTTGCACA | GAAATTCTGTCCGGTTTCAGA |
| ***EPHA3*** | **3.1** | GTCCATTAACTCAGAGAACCTTGC | TCTTTGTAAACTGATGCTCTCG | GGCTCTGACACCCTTATGTTG |
| ***EPHA3*** | **3.2** | CCAGGAACTCAGCTCAGAAGA | GAGTCCATGGGTACCGTGTC | TCCATTGGTTTTAGGAACTTGC |
| ***EPHA3*** | **3.3** | TAGGTCCTGTCAACAAGAAGGG | TGCCAAGTGAGTTGAGACTGAG | GATGTTGGTGCTTGTGTTGC |
| ***EPHA5*** | **5** | CAGACCAGGAATTGGTGTGATA | TGAGGGAAAATTCTCACACAGAT | CACACAGATTTCAGTCAACTTGG |
| ***EPHA5*** | **6** | GCTACGTTCTCAAAATGCCAATA | GCAGCAGAACATGAATAGAGAGC | CCCTGTACACTTCTAGCCTCCAT |
| ***EPHA5*** | **8** | AGGGGAAATTTCAGCTAAGTGTG | GCTGATATATTGCTTGCCTGATT | GTCAGATCAAGGTGAGGAAGAGA |
| ***EPHA5*** | **15** | CTAGATCTTTTCAGTGAGGCAACA | CAAAACAGATCAGAGGGTAAGCA | TCAGTGAGGCAACACTGTCC |
| ***EPHA5*** | **18** | TGGAGGTATAATTTTAGAGCCTAACC | GATTACAAATTCTGTGGCTGAGG | GCCAACATGAAATATTGAGGTGT |
| ***EPHA6*** | **14** | GGTTGGTTTCCTTCTGTCCTTT | AGGAAAGGGCATATAAACTTACAG | TCACCAAAGTTGATGCTTATCTTC |
| ***EPHA6*** | **3.1** | TGCTGTTCCATTGATACTTTGAG | CGAATTTCAGTGTTGAGTTTGAG | CTTTGAGTAGCTTCATTTGTATCCTT |
| ***EPHA6*** | **3.2** | AGATGAGTCCCACGGAATTAAA | GCAAGAACCCTCAATTTCTTCAT | GCCAAACCAGTATACAAAGATCG |
| ***EPHA6*** | **3.3** | GATTCCTCCTCTTTGGTTGAAGT | CGACTTTACTGAAAGCACTTTGG | TTTCATACACCTTTCCCAGGAG |
| ***EPHB2*** | **4** | AGAGATGAGATTTTCCAGCAGTG | ACCAGGGAAAGACTGGCTGTAAC | TCTGAGCTTTTCTGCAGGTAGAC |
| ***EPHB2*** | **11** | GTTCTGTGTCTGCAAGGATGAGT | GAAGCTATGTCCATCGTTCCTTT | AGTGGACATGACAGGGAACAG |
| ***EPHB2*** | **12** | GTTCTCACCACCACTCTGAAGTT | TCTTCTCTGGCTCTGTGACTCC | CATTATGAGGATGATGCAGAGC |
| ***ERBB2*** | **18** | AGCAGAAGATCCGGAAGTACAC | CTCCCTTCTCCGCTGTAACTG | GAGACTGCTGCAGGAAACG |
| ***ERBB2*** | **19** | CTAGGGTGGTGAAGGATGTTTG | GGGTCCTTCCTGTCCTCCTA | GGATGTTTGGAGGACAAGTAATG |
| ***ERBB2*** | **20** | AGGCTGGTACTTTGAGCCTTC | CAAAGAGCCCAGGTGCATAC | CTGTGGTTTGTGATGGTTGG |
| ***ERBB2*** | **21** | GTGTATGCAGATTGCCAAGGTAT | GCTCCTTGGTCCTTCACCTAA | GTATGCACCTGGGCTCTTTG |
| ***ERBB2*** | **22** | GCTGGGTGGAGTGGTGTCTA | AGCTCTCATCCTCCCTCCAG | TAGCCCATGGGAGAACTCTG |
| ***ERBB2*** | **23** | GCTACCTGCCATGATGCTAGA | AGGACCTCCCACCCTCCT | GCAGAACCTCTGGCTCAGTA |
| ***ERBB4*** | **8** | TGTGGAGCAGTAACCAAGCA | AAACCTTGTTATATAGGCCCAGTTC | CACACATTGCATTTGACTGG |
| ***ERBB4*** | **25** | CCGGTGGTTGTGCTAAAGAC | GCACATCTTTTGTGGGTATGG | GGCATCACATTGATTTGAGCTA |
| ***FGFR1*** | **4** | TCATCACTAAGGGAGCAGTGG | GGGCAGTAAGATAGGAAACAGTG | GTTCATCTGGAACTGCACTAGC |
| ***FGFR1*** | **7** | AGAAGTGCTGGGAGGTTTACAA | GTAGACTGGCCCACGAAGACT | GAGGTTTACAACCCATCACTGG |
| ***FGFR1*** | **10** | CTCTGCCATTGTTGGGAAG | CCACTAGAATAGCAAGCAAGGAA | CCTGACTAAGAATGGGAAGGAGT |
| ***FGFR1*** | **12** | GAGAATCAAGTCCCAGGGAAA | CCAGATCCCGAGATAACACATT | GCCAAAGCAGCCTCTCTTAAC |
| ***FGFR1*** | **13** | GGTAGTAGAATGGATTTCCCAGGT | GTTCCCACCCTGGCATTAC | AGGCAGGAGATGGGAGGTT |
| ***FGFR2*** | **13** | TTGCTGAATTGCCCAAGG | TTCCAGGTTGTACAAGACATGC | CTAGCAAATGAGCATGTCCAAA |
| ***FGFR2*** | **5** | AGCGAAATGATCTTACCTGTTTG | AAGAAATGTGATGTTCTGAAAGC | ACCTGTTTGGAGTTTACTCATGG |
| ***FGFR2*** | **7** | GCCACAGTGTTATTTCAAAGGTG | CCAGTTGTGGGTACCTTTAGATTC | TGACAGCCCTCTGGACAAC |
| ***FGFR3*** | **6** | GAGAACAAGTTTGGCAGCATC | CCTAGACCCAAATCCTCACG | GTACACGCTGGACGTGCT |
| ***FGFR3*** | **7** | CTCACCTGGGACAGAGGACT | GGCCGTAAGTCACAGGATTC | GGCTGAGGAGTTGGTGGT |
| ***FGFR3*** | **8** | TCACTGGCGTTACTGACTGC | CTCTACATGGTGAGCAGAGACG | CTGCAGAGAGGGCTCACAC |
| ***FGFR3*** | **9** | CATGTAGAGCCTAGGGTACTTTGG | TCTGACTGGTGGCTGTTTCA | TCATTCAATGCTGGTGGAAGT |
| ***FGFR3*** | **13** | GAGTACTTGGCCTCCCAGAA | TGAGTGTAGACTCGGTCAAACAA | AGGTGTGGGTGGAGTAGGC |
| ***FGFR3*** | **17** | GACCTGGACCGTGTCCTTAC | AAGCTCTGTGTAGCTGTCTCTCC | CCAGAGTGCTGAGGTGTGG |
| ***FGFR4*** | **13** | CAAGAACATCATCAACCTGCTT | AGTGGAGCTGGAGAGACTGAGAG | GTTAGGGTGCAGAGCCAAAG |
| ***FGFR4*** | **16** | AGGGCTCCTTCAGATTTGGT | GAGGAGGAGGACTGGAAAGTG | CTGTGGTGGGTCATGTCTGT |
| ***FLT1*** | **9** | CTGATGATCAAAGGTTTGAAATTG | GATGTTGTTACGCTGATTTTTGA | TGAGCTCAAAAACTTCAATGACC |
| ***FLT1*** | **16** | TGTATACTGCCTGGCTTAGAAGG | GAAAGAAAGAGGGTCCAACATTT | GGGTTCGCTATGATAAACCATTT |
| ***FLT3*** | **16** | ATGCCTGGCTTCTCTCATAATTT | AATTCCACTTGGGTTTGAGAGTT | TAATGCAGATTGACTCTGAGCTG |
| ***FLT3*** | **20** | GCACAGCCCAGTAAAGATAAGAG | ACCATAAATCAAAAATGCACCAC | CACCGGTACCTCCTACTGAAGTT |
| ***FLT3*** | **13 & 14** | ATTACTGAAACAGGATGTGAGAGA | CCCATTTGAGATCATATTCATATTC | TGTGAGAGATTATAATGAGTTGTCCAC |
| ***FLT3*** | **14 &15** | CTGCAGAACTGCCTATTCCTAAC | AAAGGATGGAAAAGAGAAGAAGG | AAAGCCAGCTACAGATGGTACAG |
| ***FRAP1*** | **2** | GCCGATAGCCCACAATTTAA | GATGGTACATTTAGCCCACACA | ACACTGCCTCTTTTAAACATCAGTC |
| ***FRAP1*** | **55** | TTTCCCCTTTAGGGTAGGTAGG | TGGAACCTTTTCTGCTCAAAG | GGCAGGCGTTAAAGGAATAG |
| ***IDH1*** | **4** | AATGAGCTCTATATGCCATCACTG | TTCATACCTTGCTTAATGGGTGT | GCCATCACTGCAGTTGTAGGTTA |
| ***IDH2*** | **4** | M13_GTCTGGCTGTGTTGTTGCTTG | CAGAGACAAGAGGATGGCTAGG | M13 = GTAAAACGACGGCCAGT |
| ***KDR*** | **1** | CCTCCGCGCTCTAGAGTTT | ATTCCGAGTTAGATCTGGCTTTC | CTCCCACCCTGCACTGAG |
| ***KDR*** | **17** | AAGGGAAACTAGAAGCAGGGTTA | CATTCTAATGGAGGAAGAGATGG | GCAGGGTTATAATAGGACCACTC |
| ***KDR*** | **24** | TGAACCCAGTGCTTGGTTAAGT | TGCACATCCTCATCACCTATGTA | TAGAGAGCTTCAGGACCTGTGTT |
| ***KIT*** | **2** | ATAAATAGCAGGGCAGCTTTGTC | GGCTCAGTCATCCATATGTCATC | CAGAAGATGGAACTCAGTATTGGA |
| ***KIT*** | **8** | AACTTGCTCCCTCAGGCTACT | TTCAAGTGAATTGCAGTCCTTC | GGGATTAGAGAGGGAGTGAAGTG |
| ***KIT*** | **9** | CTCACTAGGTCACCAAAGTGCTTA | TGGTAGACAGAGCCTAAACATCC | AGTATGCCACATCCCAAGTGTT |
| ***KIT*** | **14** | TTGGGACTAAGTAGTCTGATCCA | ACCTCAGAGTACCTCAGTTCATTT | CATGACCACCCTTGGGTATTT |
| ***KIT*** | **15** | AGACGGGAAATTTCTAACCTGAG | CTGCTACCATAAAGCAGAACTGG | GGTCCAGTCTATTATGTAGCAAAGG |
| ***KIT*** | **17** | GTGAACATCATTCAAGGCGTACT | GTAATGTTCAGCATACCATGCAA | AATGTGTGATATCCCTAGACAGGA |
| ***KIT*** | **18** | AGGTGATTGGGATCATCTGAGT | GGCTCTTACATTTCAGCAGGT | TCAGCAACAGCAGCATCTATAAG |
| ***KIT*** | **10 & 11** | TAACCAAGGTGAAGCTCTGAGAC | AAGCCACTGGAGTTCCTTAAAGT | GTCAGTTTGGGACTGAGTGG |
| ***KIT*** | **12&13** | ACAAATGGTCCTTCAATTCCAC | AGCAAGAGAGAACAACAGTCTGG | TCAATTCCACCACCAGCAC |
| ***MAP2K4*** | **4** | ACTGCCTAAGAACTTTGTTGCAT | ATAGAATCGAATCCTGCCATCTT | ATCCTGCCATCTTCATTTGTC |
| ***MAP2K4*** | **8** | GACGCTAGACATGGATTCCTCTA | CCATTCTTAGTGCAGTTGCAGA | CTGTCTACCCAGCTGTTGCTT |
| ***MAP2K4*** | **9** | CCCAAGCTAACCTGTGTTTAATTC | TACCAATGCTGCTAAGACCAAGT | CCTGTGTTTAATTCAAGGCTTTAC |
| ***MET*** | **14** | GATTGATTGCTGGTGTTGTCTC | AATGTCACAACCCACTGAGGTAT | TGCTGGTGTTGTCTCAATATCAA |
| ***MET*** | **16** | TGAAGCTCATAAAGGGTTTGATA | AAAACAAATTTTCAGGATTAGGC | CCATAATTTCAGTGGTAGCTGAT |
| ***MET*** | **17** | ACAAGATGCTAACTGTGTGGTTT | TTAAATGTGCATCTTTGGCTACT | TTACCATTTCATTGCTCTTCCTA |
| ***MET*** | **18** | CTTGAGCCATTAAGACCAAACTA | TTTGCATAAGAAGAGAAAACAGC | GCTTAACTAGCATTGAACAGTGG |
| ***MET*** | **19** | TCCTTCAGAAGTTATGGATTTCA | TATGAAGAAAACTGGAATTGGTG | TTCAAATACTGAAGCCACTTGTT |
| ***MET*** | **20** | AGCCAAGTTTAGTTACCAAGACC | CCAGCATTTTAGCATTACTTCAT | CCAAAAAGAAAGACATGCTGTAA |
| ***NRAS*** | **2** | AATGGAAGGTCACACTAGGGTTT | AAAGATGATCCGACAAGTGAGAG | CACACTAGGGTTTTCATTTCCA |
| ***NRAS*** | **3** | ATTTGAGGGACAAACCAGATAGG | TCCCTAGTGTGGTAACCTCATTTC | GGCAGAAATGGGCTTGAATAG |
| ***NTRK2*** | **5** | AGTGAAAGAGAGAGAGATCTGGATG | GACTGAGAGACTGAAACTTGAGAAA | GAGAGAGATCTGGATGTAAGTAAAGC |
| ***NTRK2*** | **17** | TCTCCTCTTCATGCTAAGTCAGG | GTTATGACAGCCTCAGCAAACA | GCTAAGTCAGGCAGCATCTTT |
| ***NTRK2*** | **18** | TCTGTCTCTGTTGCTTGAGACTG | AATGGATGCCTCTGGGATCT | TGCTTGAGACTGTGAAGAAGTCA |
| ***NTRK3*** | **15** | AAGGAAGGAGTCAGGCCTTATAG | TTTCTCATCCTGAGAGGAAAGTG | GCCAGTTTCTTTCCAGGAGTAG |
| ***NTRK3*** | **16** | AATAAGCCATAGGCAGGATGG | AGAGTGACAGGGTTAATGGACAA | GCAGGATGGAAAGGGAAAT |
| ***NTRK3*** | **17** | TATCGTAGGTCTCCAAAGTCAGC | TCTGGGCTGAGATAGCTCTTATG | CATCAAGAGTGCATCTATGTGTGA |
| ***PAK4*** | **4.1** | CTCCTCTGTCCCCACCTTC | GCTCATGGGATACTCGCTGT | CTCCTGCTTAGGGAGCAGA |
| ***PDGFRA*** | **12** | GTGAACGTTGTTGGACTCTACTG | GTAAAGTTGTGTGCAAGGGAAA | GTCCAGTCACTGTGCTGCTT |
| ***PDGFRA*** | **15** | TATGGTCTGCAGGACAATTCAT | GGTGGTTTGACTCTAAGTCTTGC | GCACTGAATCTGCAGACATGATA |
| ***PDGFRA*** | **17** | TCTGACCTCAGGCAATCCA | ACACTCCACTCACTGAAATCTGG | GCCTCTGCAACCTGATGATT |
| ***PDGFRA*** | **18** | AGAGCTTTCTCTCTGTTGGGAGT | CACCGAATCTCTAGAAGCAACAC | GAGAAGGCCAGCCCTTTATATC |
| ***PDGFRA*** | **19** | GCACAAGTTATTAAGAGCCCAAG | ATGGGCCTATCTCAGACACAATA | GGCCTCACACCAGGTTATCTTA |
| 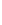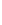***PDGFRA*** | **23** | GGCAGAATCTTGCCATACTGT | TAAACATCAACCAAGTCCTCCTC | AAGGCTTTCGTTTGTCTCTGG |
| ***PDPK1*** | **10** | CCTGACCTCAGGTGATCTGC | CGGCAGCAAAGACTACAACC | GGCTACCAGGTTTGGGTTTC |
| ***PDPK1*** | **14** | GTCATCAGCCTGTGTAGTTGCT | TAAAAATGCTGCAAGGTTTCC | AATAACCGTCACACCCACGT |
| ***PI3KCA*** | **2.1** | GTTTCTGCTTTGGGACAACCAT | CTGCTTCTTGAGTAACACTTACG | GATTCATCTTGAAGAAGTTGATGG |
| ***PI3KCA*** | **2.2** | CTCCACGACCATCATCAGG | GATTACGAAGGTATTGGTTTAGACAG | ACTTGATGCCCCCAAGAATC |
| ***PI3KCA*** | **3** | TCATCAAAAATTTGTTTTAACCTAGC | TATAAGCAGTCCCTGCCTTC | TCTACAGAGTTCCCTGTTTGC |
| ***PIK3CA*** | **4** | GCAGCCCGCTCAGATATAAAC | CTGGGCGAGAGTGAGATTCC | AAAAAGCATTTCTGATATGGATAAAG |
| ***PI3KCA*** | **5.1** | TCTTGTGCTTCAACGTAAATCC | CGGAGATTTGGATGTTCTCC | AAAATAATAAGCATCAGCATTTGAC |
| ***PI3KCA*** | **5.2** | TCTCAACTGCCAATGGACTG | CGGAGATTTGGATGTTCTCC | TTATTCCAGACGCATTTCCAC |
| ***PI3KCA*** | **6** | TAGTGGATGAAGGCAGCAAC | TTTGTAGAAATGGGGTCTTGC | TCTGAACAAAAATTCCGTGGT |
| ***PI3KCA*** | **7** | TGCCTTTTCCAATCAATCTC | AATTCCTGAAGCTCTCCCAAG | TTCCTGTTTTTCGTTTGGTTG |
| ***PI3KCA*** | **8** | GGGGAAAAAGGAAAGAATGG | TGCTGAACCAGTCAAACTCC | TGAATTTTCCTTTTGGGGAAG |
| ***PIK3CA*** | **9** | TTTGCTGAACCCTATTGGTG | TTGCAATATTGGTCCTAGAGTTC | TGGATCAAATCCAAATAAAGTAAGG |
| ***PI3KCA*** | **10** | GATTGGTTCTTTCCTGTCTCTG | CCACAAATATCAATTTACAACCATTG | TTGCTTTTTCTGTAAATCATCTGTG |
| ***PIK3CA*** | **11** | ACCTTTTGAACAGCATGCAA | TGGAAATAATGTTAAGGGTGTTTTT | TATTTCATTTATTTATGTGGAC |
| ***PIK3CA*** | **12** | AAAACACCCTTAACATTATTTCCATAG | TCTGCATGGCCGATCTAAAG | GAAGTTAAGGCAGTGTTTAGATGG |
| ***PI3KCA*** | **13** | AAACTGACCCTGATTTGTTTTTTTG | AATATTCATCTGGACCTAGAAAATTTG | GAGAAGCTCATCACTGGTACAAAATAC |
| ***PI3KCA*** | **14** | ACCTGAAACTCATGGTGGTTTTG | TGGCCTTATGAAGCAGGTATTATTT | CATTTTACAGACAAAGAAAATGAGTCC |
| ***PI3KCA*** | **15** | GAGTGTTGCTGCTCTGTGTTG | TTGAGGGTAGGAGAATGAGAGAG | TCTCATGTGAGAAAGAGATTAGCAG |
| ***PIK3CA*** | **16** | GGATTCCTAAATAAAAATTGAGGTG | CATGCATATTTCAAAGGTCAAG | TGGCTTTCAGTAGTTTTCATGG |
| ***PIK3CA*** | **17** | TTGCTTTCCTGAAGTTTCTTTTG | TCTAAGTAAGAGGAGGATATGTCAAAG | CATGTGATGGCGTGATCC |
| ***PIK3CA*** | **18** | GGGGAAAGGCAGTAAAGGTC | CATCAAATATTTCAAAGGTTGAGC | AGGAATACACAAACACCGACAG |
| ***PI3KCA*** | **19** | TCCTTATTCGTTGTCAGTGATTG | GTCAAAACAAATGGCACACG | TGCACCCTGTTTTCTTTTCTC |
| ***PIK3CA*** | **20** | CATGGTGAAAGACGATGGAC | TTACAGGCATGAACCACCAC | TGGACAAGTAATGGTTTTCTCTG |
| ***PI3KCA*** | **21.1** | TGGGGTAAAGGGAATCAAAAG | CCTATGCAATCGGTCTTTGC | TGACATTTGAGCAAAGACCTG |
| ***PI3KCA*** | **21.2** | TTGCATACATTCGAAAGACC | GGGGATTTTTGTTTTGTTTTG | TTTGTTTTGTTTTGTTTTTT |
| ***PTEN*** | **1.3** | CTCTGGCTGCTGAGGAGAAG | CAACCTGACCAGGGTTAAATG | TCCAGAGCCAAGCGGCG |
| ***PTEN*** | **2** | CATTGACCACCTTTTATTACTCCAG | AACTGTATCCCCCTGAAGTCC | TGGGGAAAACTTTCTTTTCATAAC |
| ***PTEN*** | **3** | CCATAGAAGGGGTATTTGTTGG | CAATGCTCTTGGACTTCTTGAC | AAAATCTGTCTTTTGGTTTTTCTTG |
| ***PTEN*** | **4** | CATGAAATAATTAATGTTAAACACAGC | TGCAATACTTTTTCCTAAAACACAAC | AAAGATTCAGGCAATGTTTGTTAG |
| ***PTEN*** | **5** | GCAATACATTATTTTTCTCTGGAATC | CAATTACACCTCAATAAAACTGAAGG | AGAAACCCAAAATCTGTTTTCC |
| ***PTEN*** | **6** | TTCTGATATCCTTTGTATTGATATTGC | TTGGCTTCTTTAGCCCAATG | ATGGCTACGACCCAGTTACC |
| ***PTEN*** | **7** | TGCTTGAGATCAAGATTGCAG | TGCCAGAGTAAGCAAAACACC | AAAATCGTTTTTGACAGTTTGAC |
| ***PTEN*** | **8.1** | AAATGCAACAGATAACTCAGATTGC | CACATCACATACATACAAGTCAACAACC | TTGGAGAAAAGTATCGGTTGGC |
| ***PTEN*** | **8.2** | CATACCAGGACCAGAGGAAACCT | TTTGACGCTGTGTACATTGGGT | CCAGGACCAGAGGAAACCTCA |
| ***PTEN*** | **9** | TCTTAAAGATCATGTTTGTTACAGTGC | GGGTAAAACAAGATTGGTCAGG | GTTCATCTGCAAAATGGAATAAAAA |
| ***RPS6KC1*** | **14** | GCGGCACTGTGTTGATTACTAC | GAGTTGTGGGCTCTGCTAATCT | TCTGTACTTGCTGTCCATAAATCC |
| ***RPS6KC1*** | **11.1** | ATATGCTTGAAACCGTACTCACC | GCCCATATTCATTGCATAATGTT | TCACCAGTCAGCAATAACAAGC |
| ***RPS6KC1*** | **11.2** | AGGAAGATGATGGCCAAGATAG | TAACTGGCACTGAGTCATCTGAG | CCAAGATAGCTCTCCAAAGTGG |
| ***RPS6KC1*** | **11.3** | ACAGTAAGGATAGCGCAAGTGAA | TTAGGATCTGAGCTGGGTAACAA | TGGAGACAGTGCTTCTAGGAGTT |
| ***RPS6KC1*** | **11.4** | GAGAAACACTATGCACAGGAGGA | CTCAGTTAATTGGAAGCAACAGC | GAGGATCCCAGGATGTTATTTGT |
| ***STK11*** | **1** | AGAACAATCGTTTCTGTTGGAAG | AGAGAAGGAAGGAAGACAGAACC | AATTTTGGAGAAGGGAAGTCG |
| ***STK11*** | **4** | TTCAGAGGGGTGGCTGAG | AATATCAGGACAAGCAGTGTGG | TCCAGAGCCCCTTTTCTG |
| ***STK11*** | **5 & 6** | CCTGCTGTTCCAGCAAGACT | CCAGATGTCCACCTTGAAGC | GTGTGCCTGGACTTCTGTGAC |
| ***STK11*** | **6** | TGTGCACAAGGACATCAAGC | CCATCTGCCGTATGAGTTACATT | CTGTGGCCAGAGAGGGTCT |
| ***STK11*** | **7** | AGACAGAGGTGTCCTTGAGTCC | CTCTCCACTCAGTCCTCTCAATG | TCTGTCCCTGGGGTAGAGC |
| ***STK11*** | **9** | GACATCTGTCAGGCTTGGAGT | CCACACCTTTCAGCCATGT | AGAAGCTGTCCTTGTTGCAGA |
| ***TGFBR2*** | **7** | GCACTCAGTCAGCACATGTTAAA | TCCTGCTGCCTCTGTTCTTT | TCACTATAGCAACAAGGTCAGCA |
| ***TGFBR2*** | **4.1** | GCATGAACCCACTTCCTGAC | CATGCTTCAGATTGATGTCTGAG | TTCCTGACAGTACTTACCTACCACA |
| ***TGFBR2*** | **4.2** | AGCAGAACACTTCAGAGCAGTTT | TAAGAGCCAGGAGATATGGATCA | TCAAGATCTTTCCCTATGAGGAG |
| ***TP53*** | **2** | GTCTCAGACACTGGCATGGTGT | CTTCCCACAGGTCTCTGCTAGG | GTGACCCAGGGTTGGAAG |
| ***TP53*** | **4** | AACAACGTTCTGGTAAGGACAAG | GTGAAGAGGAATCCCAAAGTTC | TGGTAAGGACAAGGGTTGG |
| ***TP53*** | **5** | AGACGCCAACTCTCTCTAGCTC | GAGCAATCAGTGAGGAATCAGAG | CCAGTTGCTTTATCTGTTCACTTG |
| ***TP53*** | **6** | ATCTACAAGCAGTCACAGCACAT | GTCAAATAAGCAGCAGGAGAAAG | CATGAGCGCTGCTCAGATAG |
| ***TP53*** | **7** | CCTGCTTGCCACAGGTCT | GATGAGAGGTGGATGGGTAGTAG | GTATGGAAGAAATCGGTAAGAGG |
| ***TP53*** | **8 & 9** | GAGCTTAGGCTCCAGAAAGGA | TTAGCTACAACCAGGAGCCATT | GACAGGTAGGACCTGATTTCCTT |
| ***TP53*** | **10** | CAAGATTGCACCATTGCAC | AATCCTATGGCTTTCCAACCTA | CAGCTGTATAGGTACTTGAAGTGCAG |
| ***TP53*** | **11** | CTTGATTTGAATTCCCGTTGTC | CAAGGGTTCAAAGACCCAAA | CCTTAGGCCCTTCAAAGCAT |

**Additional file 1: Table S1.** Thirty-nine genes selected for mutation analysis and primer details to sequence the indicated 174 exons of the selected genes. Primer sequences are in 5’ to 3’ direction.
